# Perceptive Biases in Major Depressive Episode

**DOI:** 10.1371/journal.pone.0086832

**Published:** 2014-02-18

**Authors:** Marine Naudin, Tatiana Carl, Simon Surguladze, Catherine Guillen, Philippe Gaillard, Catherine Belzung, Wissam El-Hage, Boriana Atanasova

**Affiliations:** 1 INSERM U930, Université François Rabelais de Tours, Tours, France; 2 Clinique Psychiatrique Universitaire, CHRU de Tours, Tours, France; 3 Institute of Psychiatry, King's College London, London, United Kingdom; University of Houston, United States of America

## Abstract

**Introduction:**

Alterations in emotional processing occur during a major depressive episode (MDE), and olfaction and facial expressions have implications in emotional and social interactions. To gain a better understanding of these processes, we characterized the perceptive sensorial biases, potential links, and potential remission after antidepressant treatment of MDE.

**Methods:**

We recruited 22 patients with acute MDE, both before and after three months of antidepressant treatment, and 41 healthy volunteers matched by age and smoking status. The participants underwent a clinical assessment (Mini International Neuropsychiatry Interview, Montgomery-Åsberg Depression Rating Scale, State-Trait Anxiety Inventory, Physical and Social Anhedonia scales, Pleasure-Displeasure Scale), an olfactory evaluation (hedonic aspect, familiarity and emotional impact of odors), and a computerized Facial Affect Recognition task.

**Results:**

MDE was associated with an olfactory bias concerning hedonic and emotional aspects, including negative olfactory alliesthesia (unpleasant odorants perceived as more unpleasant), facial emotion expression recognition (happy facial expressions), and in part olfactory anhedonia (pleasant odorants perceived as less pleasant). In addition, the results revealed that these impairments represent state markers of MDE, suggesting that the patients recovered the same sensory processing as healthy subjects after antidepressant treatment.

**Discussion:**

This study demonstrated that MDE is associated with negative biases toward olfactory perception and the recognition of facial emotional expressions. The link between these two sensory parameters suggests common underlying processes.

## Introduction

Depression is a major public health issue, and the main treatment used is antidepressant therapy. However, some studies have shown that the initial state is not restored after clinical remission obtained by antidepressant treatment, particularly with regard to neural mechanisms [1]. The aim of the present study is to characterize the effect of remission by antidepressant treatment on emotional biases observed in depression [2], which remain unknown. Depression is characterized by anhedonia [3], and it has been suggested that biases in the recognition of emotions could heighten interpersonal functioning impairment in depression [4]. Two sensory mechanisms have strong implications in these features: olfaction and facial expression recognition.

Anhedonia is the inability to experience pleasure from activities that are typically considered enjoyable [5]. Because olfaction evokes stronger emotional memories than other types of sensory stimuli [6], this feature might provide a suitable approach to understanding anhedonia. However, previous studies evaluating the hedonic aspect of odors in depression have reported conflicting results. Although some studies demonstrate no significant difference between depressed patients and healthy controls [7] [8] [9] [10], other studies show an overevaluation of the pleasantness of odors in depression [11]. Except for two recent reports [12] [13], most studies have not considered the valence of odor when analyzing the results, which may explain the observed inconsistencies. A previous study conducted in our laboratory [13] has shown that depressed patients perceive pleasant stimuli (with a high emotional component) as less pleasant than controls, a phenomenon called olfactory anhedonia. However, this alteration was found to be restored after remission, and we suggested that olfactory anhedonia could be a state marker of depression for high emotional component. The aim of the present study is to test whether the effect of remission by antidepressant treatment is the same on the emotions induced by different types of stimuli: odors versus faces.

Facial emotion recognition is directly implied in social interactions. Although a bias with regard to this feature has been established in major depressive episode (MDE), the nature of this condition requires additional examination. Previous studies have revealed either a generalized deficit in the recognition of all primary emotions (fear, anger, surprise, disgust, happiness, sadness, and indifference) [14] or emotion-specific abnormalities [15] [16] [17] in depression. Surguladze et al. (2004) [17] showed subtle deficits in discrimination accuracy and an identification bias of happy expressions in depressed patients; the authors suggested that this emotion-specific bias could be due to the interpersonal functioning impairment in depression.

The partial overlap between different parts of the brain (e.g., the limbic systems and the orbitofrontal cortex) that are involved in olfaction, emotion, and depression (for a review, see [18]) suggests that there is a link between the mechanisms underlying these processes. Indeed, understanding the perception of the sensory environment might be the key to elucidating the mechanisms underlying depression. In addition, the persistence (trait markers) or improvement (state markers) of these emotional biases after antidepressant treatment might reveal whether medication facilitates the recovery of sensory perception in individuals suffering from depression. This question is crucial for understanding the effects of antidepressant treatments and the status of patients during remission.

Based on the main clinical characteristics of depression and on all the above-cited studies, we made some hypotheses concerning the hedonic evaluation and emotional task. We propose that depressed patients would perceive pleasant odorants as less pleasant (olfactory anhedonia) and unpleasant odorants as more unpleasant (olfactory alliesthesia) than controls, and we predict a restoration of this impairment in patients in response to treatment. We also investigated odor familiarity because this parameter could influence the hedonic and the emotional perception of odor. We hypothesize reduced accuracy in the recognition of positive facial expressions (happy faces) in depressed patients compared to healthy controls and that this impairment would be restored in patients responding to treatment. Furthermore, we studied the relationship between the clinical state of the subjects and their olfactory and facial emotional recognition abilities.

## Methods

### Participants

At visit 1 (V1), we included 22 depressed patients within two weeks of admission to the Department of Psychiatry at University Hospital (Tours, France); these patients exhibited a current DSM-IV [3] diagnosis of acute MDE (single or recurrent episode). A total of 20 patients were treated with escitalopram, 1 patient was treated with venlafaxine, and one patient was treated with paroxetine. All 22 depressed patients were retested after clinical improvement (visit 2, V2), which occurred after an average of three months (100 days; SD = 64) of antidepressant treatment. Clinical improvement was defined by a psychiatrist who has observed a large improvement in disease symptoms and a significant reduction in the depression score evaluated with a Montgomery-Åsberg Depression Rating Scale (MADRS). A control sample of 41 age-matched healthy volunteers, with no history of mental illness, was included.

All participants were evaluated using MADRS [19], the Mini-International Neuropsychiatric Interview [20], the State-Trait Anxiety Inventory (STAI; [21]), the French translation of Physical and Social Anhedonia scales (PAS and SAS; [22] [23]), and the Pleasure-Displeasure Scale (PDS; [24]). PDS measures a subject's affective responses to pleasant, unpleasant, and neutral situations. The clusters of responses were analyzed separately. STAI was used only for the healthy controls and patients at V1.

During V1, the patients showed significantly higher state (U = 899, p<0.001) and trait (U = 901, p<0.001) anxiety inventory scores than the healthy controls. At V2, 85% of the patients had at least a 50% reduction in their MADRS score compared to V1 (Table 1).

**Table 1:** Demographic and clinical characteristics.

	Depressed patients (V1) (n = 22)	Clinically improved patients (V2) (n = 22)	Control subjects (n = 41)
Female/male ratio	16/6	16/6	24/17
Mean age, years (SD)	33.2 (11.2)	33.2 (11.2)	34 (11)
Age range, years	19–51	19–51	22–59
Smoker/nonsmoker ratio	16/6	16/6	20/21
MADRS, mean score (SD)	37.1 (6.6)	11.3 (9.2)	1.1 (2.2)
AIS (state), mean score (SD)	57.7 (11)	-	27.2 (5.5)
AIS (trait), mean score (SD)	62.7 (7.9)	**-**	33.4 (6.3)
**MINI 5.0.0**			
MDE, current episode	22	-	0
MDE, lifetime	11	-	0
Suicidal risk, last month	20	-	0
(Hypo)-mania, lifetime	2	-	0
Panic disorder, lifetime	3	-	0
Agoraphobia, current episode	4		0
GAD, last 6 months	4	-	0
OCD, last month	1	-	0
PTSD, last month	3	-	0
Alcohol abuse, last 12 months	0	-	0
Cannabis abuse, last 12 months	0	-	0
Psychotic disorder, lifetime	0	-	0
Eating disorders, last 3 months	0	-	0

*MADRS, Montgomery-Åsberg Depression Rating Scale; AIS, Anxiety Inventory Scale; MINI 5.0.0, Mini-International Neuropsychiatric Interview version 5.0.0; MDE, Major Depressive Episode; OCD, Obsessive-Compulsive Disorder; PTSD, Post-traumatic Stress Disorder;*

*GAD, Generalized Anxiety Disorder.*

### Procedure

The present study is a monocenter, prospective, longitudinal observational study. Approval from the local ethics committee board (CPP Tours Ouest-1, France) was obtained, and the study was conducted in accordance with the Good Clinical Practice procedures and the current Declaration of Helsinki.

The experimental procedure was clearly explained to all the participants, and written informed consent was obtained prior to testing. The participants were informed of the option to discontinue testing at any time. The exclusion criteria comprised possible brain damage, major medical problems, current substance abuse, allergy, current cold, or any alteration of the sense of smell. All subjects were selected based on the absence of anosmia to the odorants used in the study. Smokers were instructed not to smoke for at least 30 min prior to testing.

### General design

Before testing began, all the tasks were explained to the participants, and a brief training session was performed. First, we assessed the participants with clinical scales, and the subjects were then asked to evaluate the hedonic aspect, familiarity, and emotional impact of odors (sensory tests); the participants then performed the facial expression recognition task (emotional task). The different tasks lasted approximately 1 hour and were presented in the same order for all participants. However, the presentation order of the odorants and the emotional stimuli was balanced across the stimuli and for all subjects yet was identical for the groups. For all the sensory experiments, the odorant solutions were prepared with distilled water, and the solutions were poured into 60-ml brown glass flasks (10 ml per flask); each flask was assigned a random three-digit number. The subjects were not limited with regard to the time allowed for sniffing. Indeed, previous experiments have shown that each individual optimizes their parameters of sniffing to obtain the maximum sensitivity [25]. However, a 30-second interval between samples was imposed to prevent olfactory adaptation.

### Hedonic aspect, familiarity, and emotional impact of odors

The subjects successively smelled eight different odorants and were asked to evaluate the pleasantness, familiarity, and intensity of the emotion of the perceived odorant stimulus on a 10-cm linear scale labeled as follows at each end: highly unpleasant and highly pleasant, unfamiliar odor and very familiar odor, and weak intensity of the emotion and strong intensity of the emotion. The resulting response was expressed in a score ranging from 0 to 10. Before evaluating the intensity of the evoked emotion, the subject selected one of the following emotions: happiness, surprise, disgust, fear, sadness, anger, or neutral (no emotion). The intensity was not evaluated when the last response was chosen. The eight studied odorants were as follows: four odors were pleasant (vanillin at 3 g/l, 2-phenylethanol [rose] at 12.25 ml/l, (E)-cinnamaldehyde [cinnamon] at 0.25 ml/l, and benzaldehyde [bitter almond] at 0.25 ml/l); two odors were neutral (eugenol [clove] at 0.25 ml/l and 1-octen-3-ol [mushroom] at 0.05 ml/l); and two odors were unpleasant (hexanoic acid [mold] at 1.6 ml/l and butyric acid [old cheese] at 0.12 ml/l) [13] [26] [27] [28]. All the odorant compounds were supplied by Fisher Scientific Bioblock (France) and Sigma (Illkirch, France). These compounds are soluble in water, and the selected concentrations were iso-intense.

### Facial expression recognition task

All participants underwent a computerized facial expression recognition (FAR) task [29] that consisted of viewing randomized pictures of 10 facial identities, each displaying an expression of happiness, sadness, anger, or fear [17]. Each face was presented twice during the task (for 500 and 2000 ms), with an inter-stimulus interval of 1500 ms. The experiment included four runs, with one run per emotion (happy versus neutral faces, sad versus neutral faces, anger versus neutral faces, and fear versus neutral faces). In each run, one face was presented, and the participants were asked to label each facial expression as emotional or neutral by moving a computer joystick to the left or right, respectively. Before testing, all the participants performed a practice trial to ensure their ability to perform the task.

### Statistical analysis

The statistical analyses were performed using non-parametrical tests because the Levene test for the homogeneity of variances revealed unequal variance for the majority of the variables and the normal distribution of the data was not always validated (Kolmogorov-Smirnov test).

A Mann-Whitney unpaired test was used to compare the patients at V1 versus the controls and the patients at V2 versus the controls, and a Wilcoxon paired test was used to compare the patients at V1 versus the patients at V2. These two tests were performed with Bonferroni correction (α* = α/k, where α = 0.05 and k is the number of the comparisons performed; i.e., α* = 0.025) and were used to compare the scores of the clinical evaluations (STAI, MADRS, PAS, SAS, and PDS) and olfactory measures (odor pleasantness response, odor familiarity level, and emotional intensity evoked through perceived odor). To study the hedonic responses, we combined the odorants into three groups (pleasant, unpleasant, and neutral) to enhance the statistical reliability. The same regrouping was used when the odor familiarity responses were studied.

A z test with Bonferroni correction (α = 0.025) was used to compare the number of citations for each emotion (olfactory test) (patients at V1 versus controls, patients at V2 versus controls, and patients at V1 versus patients at V2). For each emotion, the sum of citations was calculated for all eight odorants.

Concerning the FAR task, the raw data were transformed into measures of accuracy and response bias according to the two-high threshold theory [30]. The discrimination accuracy *Pr* was calculated for the four separate subsets of emotions (targets) versus neutral faces (distractors) [*Pr* = (hits+0.5/targets+.01)−(false alarms+0.5/distractors+1)]. The response bias *Br* was computed according to false-alarm scores (i.e., the tendency to label a neutral face as emotional) [*Br* = (false alarms+0.5/distractors+1)/(1−*Pr*)]. An ability to accurately discriminate among emotions was indicated by high accuracy values; higher response bias scores would indicate a tendency to misidentify a neutral face as emotional.

Comparison of the *Pr* scores of all emotions for each group of subjects was performed using Friedman's paired test with Bonferroni correction (α = 0.008). The post-hoc Nemenyi procedure permitted two-by-two comparisons of the *Pr* score of the different emotions.

To compare the *Pr* and *Br* scores for each emotion between the three groups, the Mann-Whitney test with Bonferroni correction (α = 0.025) was used to compare the patients at V1 versus the controls and the patients at V2 versus the controls. The Wilcoxon paired test with Bonferroni correction (α = 0.025) was applied to compare the results among the patients at V1 versus the patients at V2.

For the Mann- Whitney unpaired test, the Wilcoxon paired test, and the z test, the 0.05 level was taken to indicate a marginal effect.

The Spearman correlation coefficient was used to study the relationship between the clinical subjects' state and their olfactory and facial recognition performances. The Spearman coefficient was calculated for the two patient groups and the significant results obtained in the different tests and scales. This last statistical analysis was performed at α = 5%. All the statistical analyses were performed using XLSTAT®-Pro, release 5.2.

## Results

### Clinical measures

During V1 and V2, the patients showed significantly higher social (V1: U = 783, p<0.001; V2: U = 750, p<0.001) and physical (V1: U = 719, p<0.001; V2: U = 689, p<0.001) anhedonia scores than the healthy controls. A trend was observed between the patients at V1 and V2 for the social (V = 176, p<0.05) anhedonia score but not for the physical (V = 162.5, p = 0.25) anhedonia score (Table 2).

**Table 2:** Clinical scale scores.

	Physical and Social Anhedonia Scale		Pleasure-Displeasure Scale		
	*Physical anhedonia*	*Social anhedonia*	*Pleasure Score*	*Displeasure score*	*Neutral score*
**Depressed patients (V1)**	21.7 (9.6)^*####^	16.7 (6.6)^*####^	7.2 (0.8)^**^	2.9 (0.6)^**##^	5.0 (0.4)^##^
**Clinically improved patients (V2)**	19.0 (8.3)^tttt^	14.8 (6.3)^tttt^	6.8 (0.8)	3.2 (0.7)	5.2 (0.5)
**Control subjects**	11.7 (6.5)	7.6 (4.3)	7.1 (0.4)	3.2 (0.5)	5.3 (0.4)

Comparison of the mean (SD) of physical and social anhedonia scores and pleasure-displeasure scores among depressed patients (n = 22), clinically improved patients (n = 22), and controls (n = 41).

Patients at V1 versus patients at V2 (Wilcoxon test: ^*^≤0.05, ^**^≤0.025).

*Patients at V1 versus controls (Mann-Whitney test: ^##^≤0.025, ^####^≤0.001).*

*Patients at V2 versus controls (Mann-Whitney test: ^tttt^≤0.001).*

*The level of significance was set at p = 0.025 to avoid error due to multiple comparisons; a 0.05 level indicates a marginal effect.*

Regarding the pleasure-displeasure scale results, we observed a significant difference between the patients at V1 and the controls for displeasure (U = 291, p<0.025) and neutral (U = 283, p<0.025) responses and between the patients at V1 and at V2 for displeasure (V = 54, p<0.025) and pleasure (V = 167, p<0.025) responses. However, no significant difference between the patients at V2 and the controls was observed for displeasure (U = 443, p = 0.91), pleasure (U = 373, p = 0.26), or neutral (U = 390, p = 0.38) responses (Table 2).

### Olfactory parameters

#### Hedonic and familiarity aspects

Concerning the hedonic evaluation, the results demonstrated a significant difference between the patients at V1 and the controls for unpleasant (U = 273, p = 0.01) but not for neutral odorants (U = 405, p = 0.5); the difference was only tendentious for the pleasant odorants (U = 317, p = 0.05). No significant differences were observed between the patients at V1 and at V2 for pleasant (V = 77, p = 0.11) and neutral odorants (V = 119, p = 0.93), whereas a significant difference was observed between the patients at V1 and at V2 for unpleasant stimuli (V = 36, p<0.01). No significant differences were shown between the patients at V2 and the controls for pleasant (U = 422, p = 0.68), unpleasant (U = 474, p = 0.75), and neutral (U = 430, p = 0.76) stimuli (Table 3).

**Table 3:** Odor hedonic scores.

	Pleasant odorants	Neutral odorants	Unpleasant odorants
**Depressed patients (V1)**	5.0 (1.6)*^#^*	3.5 (2.4)	0.7 (0.6)^***###^
**Clinically improved patients (V2)**	5.5 (1.6)	3.5 (2.0)	1.4 (1.1)
**Control subjects**	5.8 (1.2)	3.9 (2.1)	1.3 (1.0)

Comparison of the mean of odor hedonic scores (SD) of pleasant, neutral, and unpleasant odorants among depressed patients (n = 22), clinically improved patients (n = 22), and controls (n = 41).

Patients at V1 versus patients at V2 (Wilcoxon test: ^***^≤0.01).

*Patients at V1 versus controls (Mann-Whitney test: ^#^≤0.05, ^###^≤0.01).*

*The level of significance was set at p = 0.025 to avoid error due to multiple comparisons; a 0.05 level indicates a marginal effect.*

There were no significant differences concerning the evaluation of the familiarity of positive (patients at V1 versus controls, U = 500, p = 0.5; patients at V2 versus controls, U = 500, p = 0.5; patients at V1 versus V2, V = 109, p = 0.6), negative (patients at V1 versus controls, U = 358, p = 0.18; patients at V2 versus controls, U = 479, p = 0.70; patients at V1 versus V2, V = 74, p = 0.09), and neutral (patients at V1 versus controls, U = 460, p = 0.9; patients at V2 versus controls, U = 548, p = 0.2; patients at V1 versus V2, V = 63, p = 0.04) odorants.

#### Emotional impact of odors and their intensity

**Figure 1:**
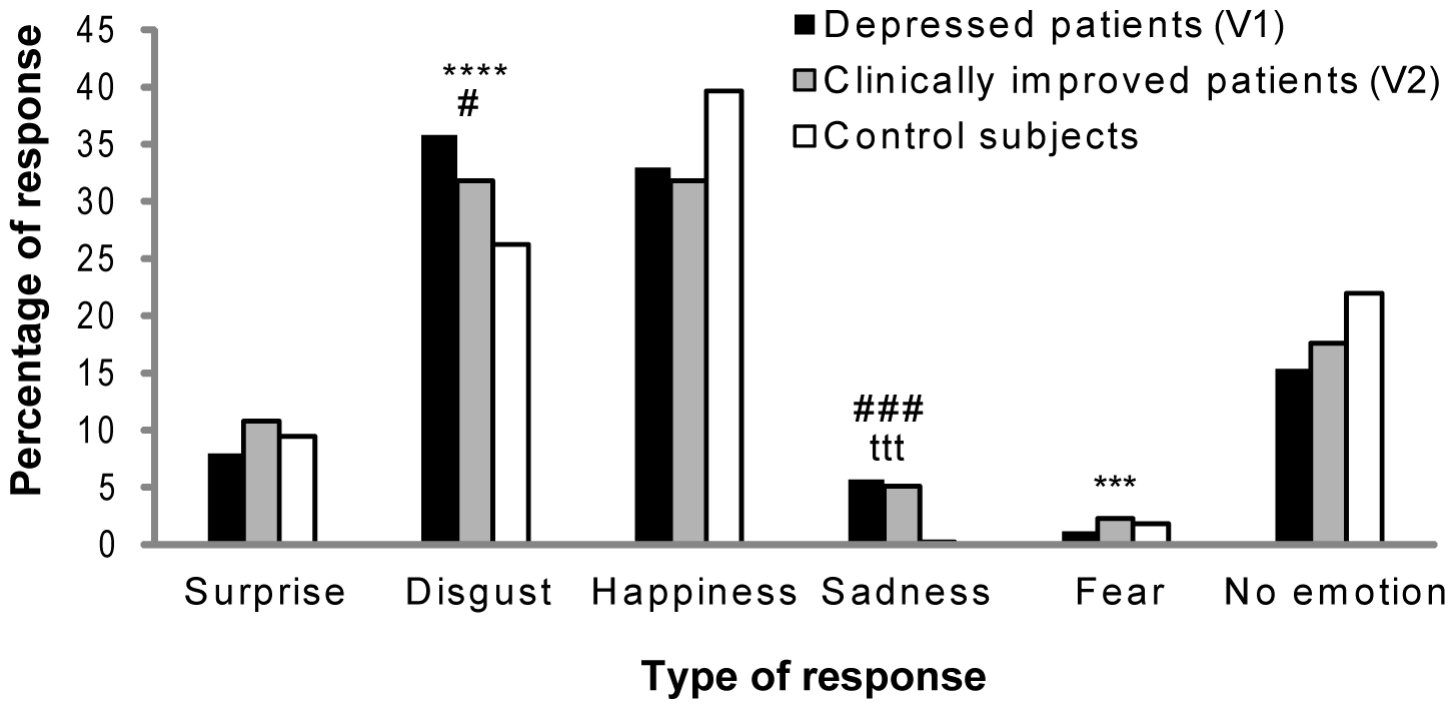
Emotional responses to odors. Between-groups comparison of the emotional responses to odors (z test). Patients at V1 versus patients at V2 (^***^≤0.01, ^****^≤0.001); patients at V1 versus controls (^#^≤0.05, ^###^≤0.01); patients at V2 versus controls (^ttt^≤0.01). The level of significance was set at p = 0.025 to avoid error due to multiple comparisons; a 0.05 level indicates a marginal effect.

Regarding the emotional impact of odors, the z test demonstrated no significant differences between the patients at V1 and the controls with regard to surprise (z = −0.58, p = 0.57), happiness (z = −1.50, p = 0.13), fear (z = −0.64, p = 0.53), and neutral (z = −1.86, p = 0.06) citations. In contrast, a significant difference between these two groups was demonstrated for a sadness citation (z = 3.04, p<0.01), and a marginal difference was observed for disgust (z = 2.20, p = 0.028) (Figure 1). Moreover, no significant differences were observed between the patients at V2 and the controls for surprise (z = 0.47, p = 0.64), happiness (z = −1.77, p = 0.08), fear (z = 0.33, p = 0.74), and neutral (z = −1.18, p = 0.24) citations. However, our results did show a significant difference between the patients at V2 and the controls with regard to sad citations (z = 2.85, p<0.01). Lastly, the z test revealed no significant differences between the patients at V1 and at V2 with regard to surprise (z = −0.92, p = 0.36), happiness (z = 0.23, p = 0.82), sadness (z = 0.24, p = 0.81), and neutral (z = −0.58, p = 0.57) citations. Significant differences were observed only for disgust (z = 4.49, p<0.001) and fear (z = 2.63, p<0.01). Anger was not analyzed because there were not enough citations (V1, 1 citation; V2, 1 citation; controls, 2 citations).

Concerning the intensity of odor emotion, we observed a significant difference only for disgust between the patients at V1 and the controls (U = 3368, p<0.025) and between the patients at V1 and at V2 (V = 1454, p<0.01), with no significant difference observed between the patients at V2 and the controls (U = 2115, p = 0.22). Additionally, no significant differences were observed for the intensity perception of surprise (patients at V1 versus controls, U = 222, p = 0.91; patients at V2 versus controls, U = 304, p = 0.86; patients at V1 versus V2, V = 117, p = 0.97), happiness (patients at V1 versus controls, U = 4326, p = 0.11; patients at V2 versus controls, U = 3992, p = 0.30; patients at V1 versus V2, V = 898, p = 0.58), sadness (patients at V1 versus controls, U = 6, p = 0.87; patients at V2 versus controls, U = 9, p = 0.2; patients at V1 versus V2, V = 13, p = 0.15), or fear (patients at V1 versus controls, U = 8, p = 0.5; patients at V2 versus controls, U = 14, p = 0.19; patients at V1 versus V2, V = 1, p = 0.42).

### Facial expression recognition task

#### Response accuracy

**Figure 2:**
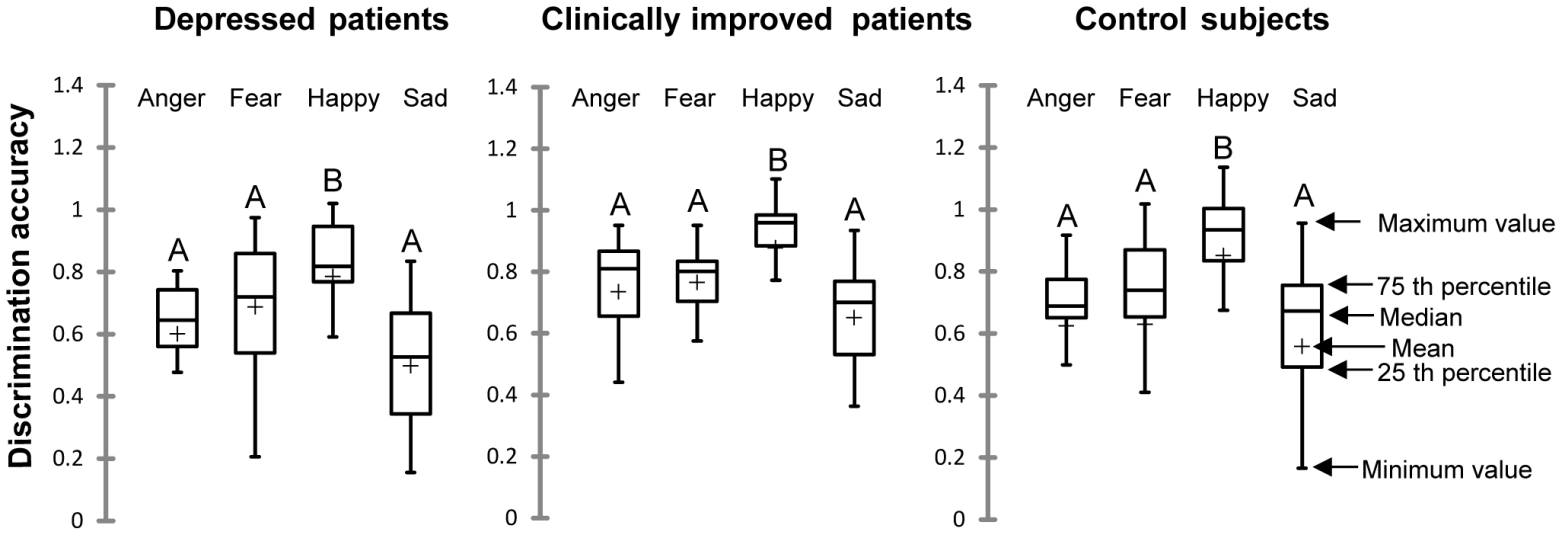
Discrimination accuracy scores of all emotions faces. Comparison of discrimination accuracy scores of all emotions (facial expression recognition task) for each group of subjects. For each group of subjects, values with the same letters are not significantly different at α = 0.008: significance level Bonferroni corrected (Nemenyi procedure).

When the groups were analyzed separately, the results demonstrated a significant difference among the ability to accurately discriminate emotions in the depressed patients at V1 (Q = 23.91, p<0.001), clinically improved patients at V2 (Q = 30.82, p<0.001), and the controls (Q = 47.93, p<0.001). The multiple comparison tests showed that the response accuracy for “happiness” was higher than for the other emotions in each group (Figure 2).

**Figure 3:**
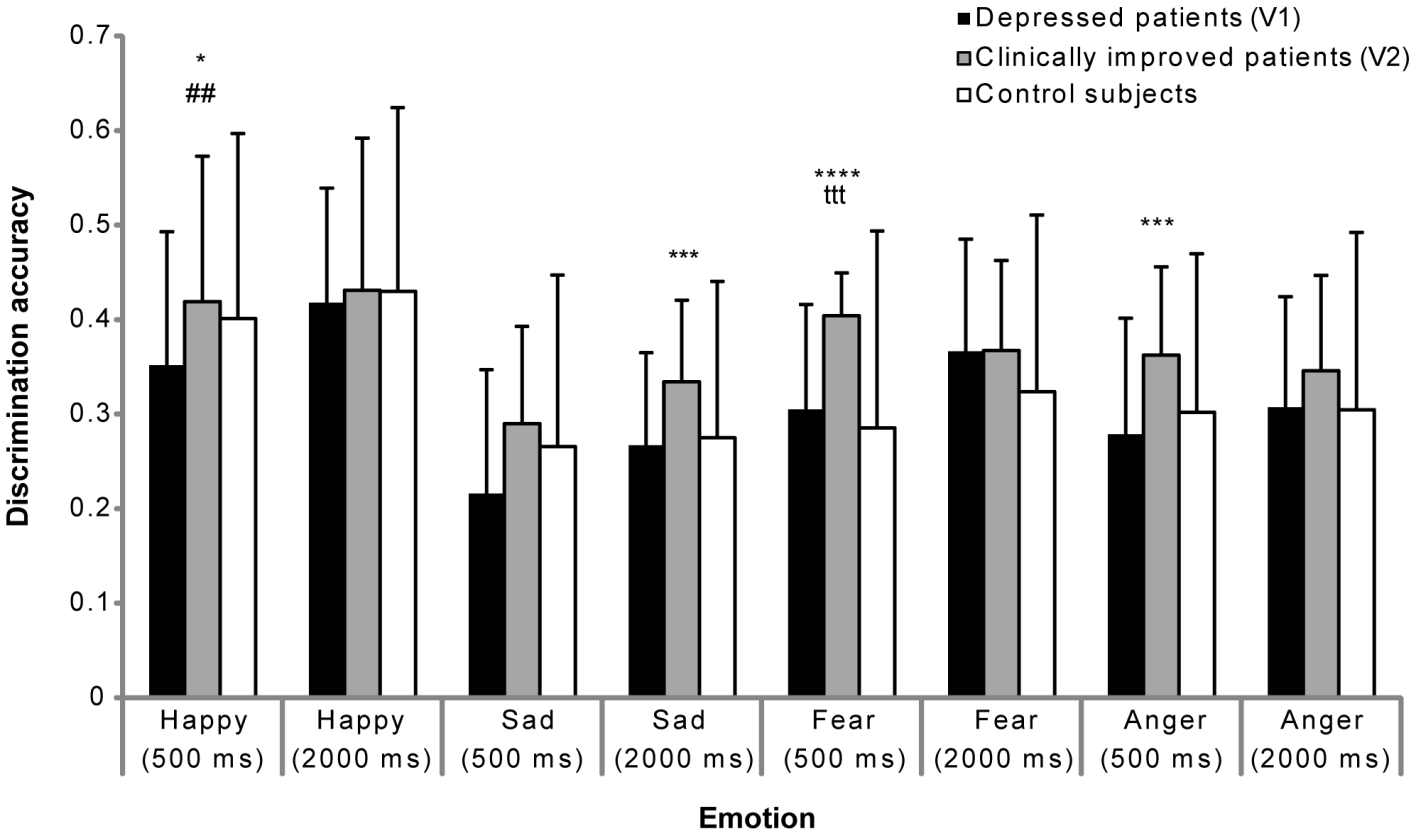
Discrimination accuracy of each emotional face. Between-groups comparison of mean (SD) discrimination accuracy of emotional faces (presented for 500 ms and 2000 ms). Patients at V1 versus patients at V2 (Wilcoxon test; ^*^≤0.05, ^***^≤0.01, ^****^≤0.001); patients at V1 versus controls (Mann- Whitney test; ^##^≤0.025); patients at V2 versus controls (Mann-Whitney test; ^ttt^≤0.01). The level of significance was set at p = 0.025 to avoid error due to multiple comparisons; a 0.05 level indicates a marginal effect.

When the accuracy to discriminate emotions was compared between the patients at V1 and the controls, the Mann-Whitney test showed a significant difference only for happy expressions when the faces were presented for 500 ms (U = 287, p<0.025), which was not the case for 2000 ms (U = 343, p = 0.12) (Figure 3). The results demonstrated a lower response accuracy for depressed patients at V1 compared to these patients at V2 for anger (V = 41.5, p<0.01) and fear (V = 23, p<0.001) faces and a tendency for happy faces (V = 45, p = 0.026) when they were presented for 500 ms. Moreover, the results showed a lower response accuracy for the depressed patients at V1 compared to V2 for sad (V = 34, p<0.01) faces when they were presented for 2000 ms (Figure 3).

#### Response bias

The results showed no significant differences among the three groups concerning anger (for 500 ms, patients at V1 versus controls, U = 479, p = 0.70, patients at V2 versus controls, U = 353, p = 0.16; at 2000 ms, patients at V1 versus controls, U = 456, p = 0.95, patients at V2 versus controls, U = 326, p = 0.07, patients at V1 versus V2, V = 184, p = 0.07), fear (for 500 ms, patients at V1 versus controls, U = 470, p = 0.80, patients at V2 versus controls, U = 380, p = 0.30, patients at V1 versus V2, V = 166, p = 0.21; for 2000 ms: patients at V1 versus controls, U = 388, p = 0.36, patients at V2 versus controls, U = 391, p = 0.39, patients at V1 versus V2, V = 127, p = 1), and happy faces (for 500 ms, patients at V1 versus controls, U = 430, p = 0.76, patients at V2 versus controls, U = 376, p = 0.28, patients at V1 versus V2, V = 130, p = 0.36; for 2000 ms, patients at V1 versus controls, U = 390, p = 0.38, patients at V2 versus controls, U = 404, p = 0.50, patients at V1 versus V2, V = 113, p = 0.95). A significant difference was observed between the patients at V1 and at V2 with regard to the series of sad versus neutral stimuli at 2000 ms (V = 194, p<0.01), and a marginal difference was revealed at 500 ms (V = 173, p = 0.048). Indeed, the results showed a more liberal (higher) response bias in the patients at V1 compared to V2 for sad faces. Thus, the patients at V1 had a greater tendency to label neutral faces as sad compared to clinically improved patients at V2.

### Correlations

At V1, a negative significant correlation was observed between the MADRS score and the response accuracy for sad faces presented for 2000 ms (r = −0.57, p<0.01). Three significant correlations were also identified for patients at V2. A significant negative correlation was demonstrated between the displeasure score and the response accuracy for happy faces presented for 500 ms (r = −0.48, p<0.05). Significant positive correlations were also observed between the odor intensity of disgust and the response accuracy of fear (r = 0.69, p<0.001) and sadness (r = 0.45, p<0.001) when the faces were presented for 500 ms.

## Discussion

The main goal of the present study was to characterize perceptive biases (i.e., olfactory and facial emotional biases) in major depression and the effect of remission on these biases. Our results show that the emotional biases observed in depressed patients prior to antidepressant treatment appears to be restored or is in the process of being restoring.

At first, we have demonstrated hedonic olfactory biases in depressives which are being restore after antidepressant treatment. Thus, our results support our first hypothesis that pleasant stimuli were perceived as less pleasant by patients with acute MDE compared with healthy controls (olfactory anhedonia). Besides, unpleasant stimuli were perceived as more unpleasant by depressive patients before antidepressant treatment. In addition, a tendency to perceive pleasant stimuli as less pleasant was also observed in patients with acute MDE compared to healthy controls (olfactory anhedonia). These results partially validate our first hypothesis and are consistent with previous findings, which have demonstrated that, when two odorants with opposite valences are presented to depressed patients, these individuals evaluate the unpleasant odorant as being significantly more unpleasant than do controls [12]. In addition, no difference was observed among the three groups for neutral stimuli (i.e., eugenol and 1-octen-3-ol), which correspond to non-emotional odors. All these results are consistent with the existence of a bias in the perception of emotions conveyed by odors in patients with major depression prior to antidepressant treatment. This phenomenon is confirmed by the fact that depressed patients before treatment cite sadness significantly more frequently than controls and they also have a tendency to cite disgust more than controls. Croy (2011) [31] described that disgust, happiness, and anxiety constitute the main emotions that can be elicited through the olfactory channel. Additionally, Bensafi et al. (2002) [32] showed that healthy subjects more frequently evoke verbally disgust and happiness than any other emotions. Our results are partly in agreement with these previous results, particularly concerning citations of disgust. From a behavioral point of view, olfactory disgust has been closely associated with nutrition, and this parameter might also be associated with the eating disorders frequently observed in MDE. Furthermore, these results could also be associated with the frequent experience of disgust in depression, with regard to both social interactions and directed toward self [33]. Taken together, our results suggest a negative potentiation and a positive attenuation (olfactory anhedonia) at the olfactory level.

Our results further demonstrated that antidepressant treatment can correct these two biases for the hedonic perception of odors. Consequently, these results are consistent with a preliminary study from our laboratory, which suggested that “olfactory anhedonia” for high emotional odorants is a state marker of MDE [13]. These results suggest that patients recover the same olfactory processing as healthy subjects after antidepressant treatment. The neuroanatomic hypothesis might explain the restoration of olfactory deficits during the second session when the patients were clinically improved. Indeed, some studies have demonstrated that orbitofrontal cortex is implied in the hedonic aspect of odors [34] [35]. Moreover, the amygdala is involved in the detection of emotional signals. In previous studies, a decrease in activity in the orbitofrontal cortex and hyperactivation of the amygdala [36] [37] were observed in depression, leading to hyperactivation in response to negative stimuli. The fact that antidepressant treatment normalizes the abnormal activation of the amygdala and orbitofrontal cortex could explain the results obtained in our study [37] [38] [39]. Another explanation of the restoration of olfactory function after antidepressant treatment can be found in a potential stimulation of neurogenesis by antidepressant drugs. Indeed, Negoias et al. (2010) [40] have demonstrated a reduction in olfactory bulb volume in patients with major acute depression compared to healthy controls, which was significantly correlated with the depression score. This alteration could explain olfactory dysfunction in depression. Moreover, authors have suggested that this alteration could be related to reduce neurogenesis in the olfactory bulb and could be resolved by the stimulation of neurogenesis by antidepressants, as has been shown at the level of the hippocampus.

Our results also showed a negative recognition bias concerning the response accuracy of happy faces for shorter presentations while preserving the response accuracy for negative stimuli (i.e., anger, fear, and sad faces). The results suggest a positive attenuation and emotional-specific deficit toward positive emotions (happy faces) rather than a generalized deficit in the recognition of emotions for depressed patients (V1) compared to healthy controls. This result is consistent with previous studies reporting deficits in the recognition of positive facial expressions [15] [16]. The impairment in the discrimination accuracy for happy faces could be explained by dysfunctional and maladjusted cognitive schema in depression [41]. Indeed, depressed patients wrongly interpret reality, and the correlation results showed that, when the displeasure score is high, the less depressed individuals were able to discriminate happy faces, thereby confirming this tendency (noted that the correlation analysis was performed without Bonferroni correction). These deficits disappeared when the faces were presented for a longer time (2000 ms); indeed, in everyday life, emotional signals appear for only a short time. It has been demonstrated that healthy volunteers are able to discriminate stimuli presented for a short duration [42], and impairment of rapid presentations of emotional expressions in depression could be due to a general slowing of cognitive processes. These results highlight the importance of different presentation durations to understand the subtle impairments in the perception of emotional faces during MDE.

Moreover, no response bias was observed in the depressed patients at V1 compared to the healthy controls. However, after treatment, the patients were significantly more conservative (i.e., had a smaller response bias) than before treatment for a sad emotion. These results suggest a modification in cognitive functioning after treatment, with depressed patients consequently selecting fewer negative stimuli (sad facial expressions) after clinical improvement. These results suggest that the dysfunctional cognitive schema observed in depression can recover. Thus, our observations revealed that impairments in response accuracy disappear in remission, a result that is consistent with the literature [43] [44]. These previous authors have reported impairments in the recognition of sad and happy faces between depressed and healthy controls, but these deficits were reported to vanish after remission, suggesting a state deficit of emotion processing during depression. Indeed, our results confirm a facial expression recognition bias as a potential state marker of depression. Thus, patients appear to recover the same facial expression recognition processing as healthy subjects after antidepressant treatment, suggesting that medication could allow patients to recover their previous sensory perception concerning these two types of stimuli.

Furthermore, only two significant correlations between the emotions perceived through olfaction and facial expression recognition were demonstrated. Indeed, more odors were perceived as disgusting by the clinically improved patients (V2), and more of these patients exhibited a higher response accuracy for fear and sad faces presented for a short duration (note that the correlation analyses were performed without Bonferroni correction). These results suggest common underlying processes between these two perception mechanisms. Implication of the amygdala in olfactory emotional processing [45] [46] and the detection of both threat and sad [37] stimuli could explain these results. Additional studies, including neuroimaging investigations, are needed to confirm these results.

Some limitations of this study must be considered. First, confirmation of the specificity and sensitivity of the olfactory and emotional tests in a larger sample, including several age ranges, is needed to create standardized tools. Second, the effects of different antidepressant treatments and other therapeutic methods on olfactory perception should be tested. Nevertheless, previous studies on olfaction have not identified any effects of usual psychotropic medication [47] [48]. Moreover, some of the patients included in this research exhibited a long history of disease and/or were simultaneously treated with several drugs. Therefore, the potential effects of chronic medication and the synergy of multiple drug use cannot be excluded. Moreover, in the patient population, we combined subjects with a single depressive episode and subjects with recurrent episodes, which added heterogeneity; thus, it would be relevant to study each group separately in future works. Lastly, even though all the participants were asked not to smoke for at least 30 min before testing, a majority of them were smokers, which could influence olfactory perception [49]. This point must be controlled in future studies.
